# Supplementary material for: Discovering Rare Genes Contributing to Cancer Stemness and Invasive Potential by GBM Single-Cell Transcriptional Analysis
Source: Cancers (Basel). 2019 Dec 16;11(12):2025. doi: 10.3390/cancers11122025 (PMC6966673; doi:10.3390/cancers11122025)
Supplement: Supplementary file 1 [file cancers-11-02025-s001.zip › cancers-666237-Supplementary figures.docx]

Supplementary Figures:

Discovering Rare Genes Contributing to Cancer Stemness and Invasive Potential by GBM Single-Cell Transcriptional Analysis

Lin Pang ^†^, Jing Hu ^†^, Feng Li, Huating Yuan, Min Yan, Gaoming Liao, Liwen Xu, Bo Pang, Yanyan Ping, Yun Xiao * and Xia Li *


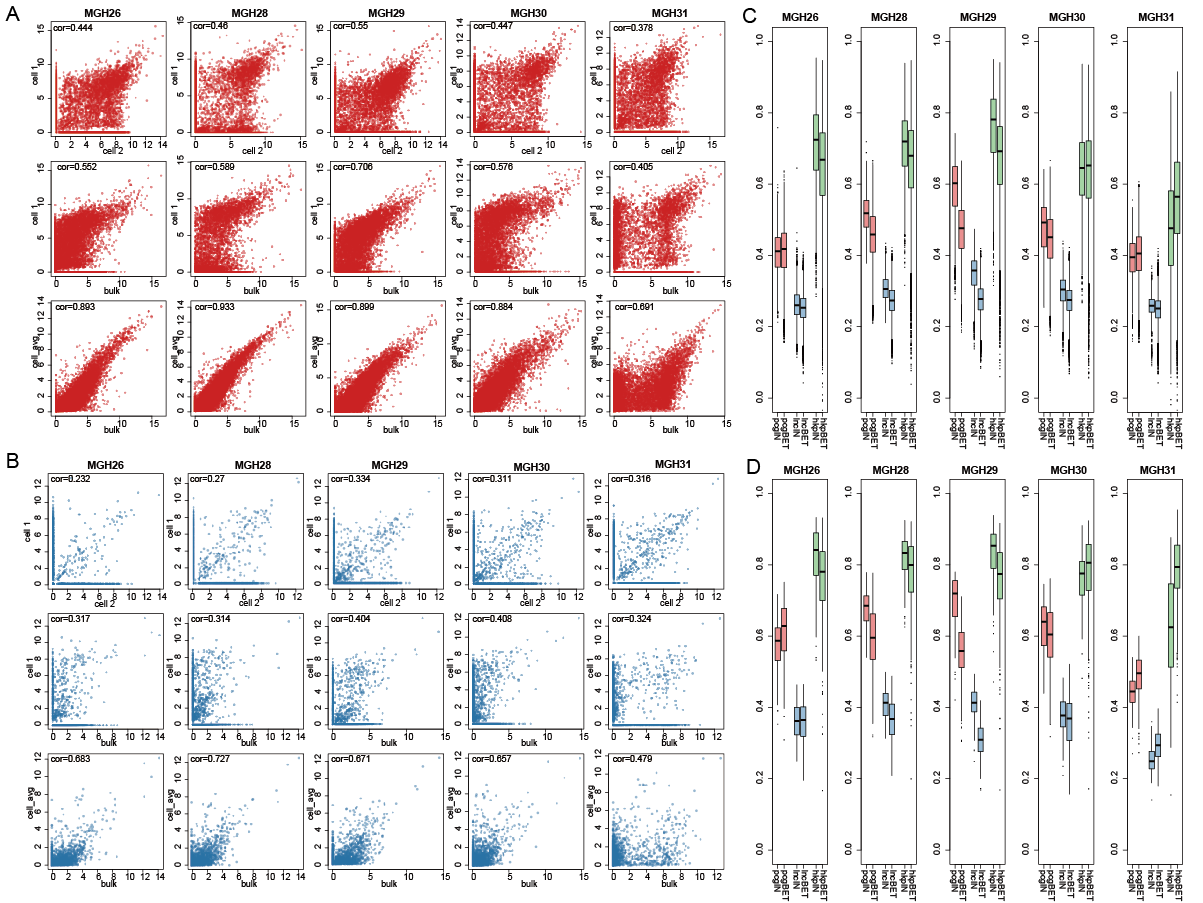


**Figure S1.** Correlation analysis across single cells and bulk samples. (A-B) Expression correlation between two randomly selected single cells (upper panel), between one randomly selected single cell and bulk sample (middle panel) and between average of single cells and bulk sample (lower panel) based on PCGs (A) and lncRNAs (B). (C) Distributions of correlation across single cells within each individual and between different individuals based on expression levels of PCGs (lightred), lncRNAs (lightblue) and housekeeping genes (lightgreen). (D) Distributions of expression correlation between single cells and bulk sample from the same individual and from different individuals. Overall, cells within the same patient showed higher correlation than cells from different patients except those from MGH31. We observed the same phenomenon when calculating correlation between cells and bulk samples. Moreover, correlation between average of single cells and corresponding bulk sample from MGH31 was substantially lower than that from other patients. We suspected that cells from MGH31 were potentially contaminated and thus we removed them from further analyses.


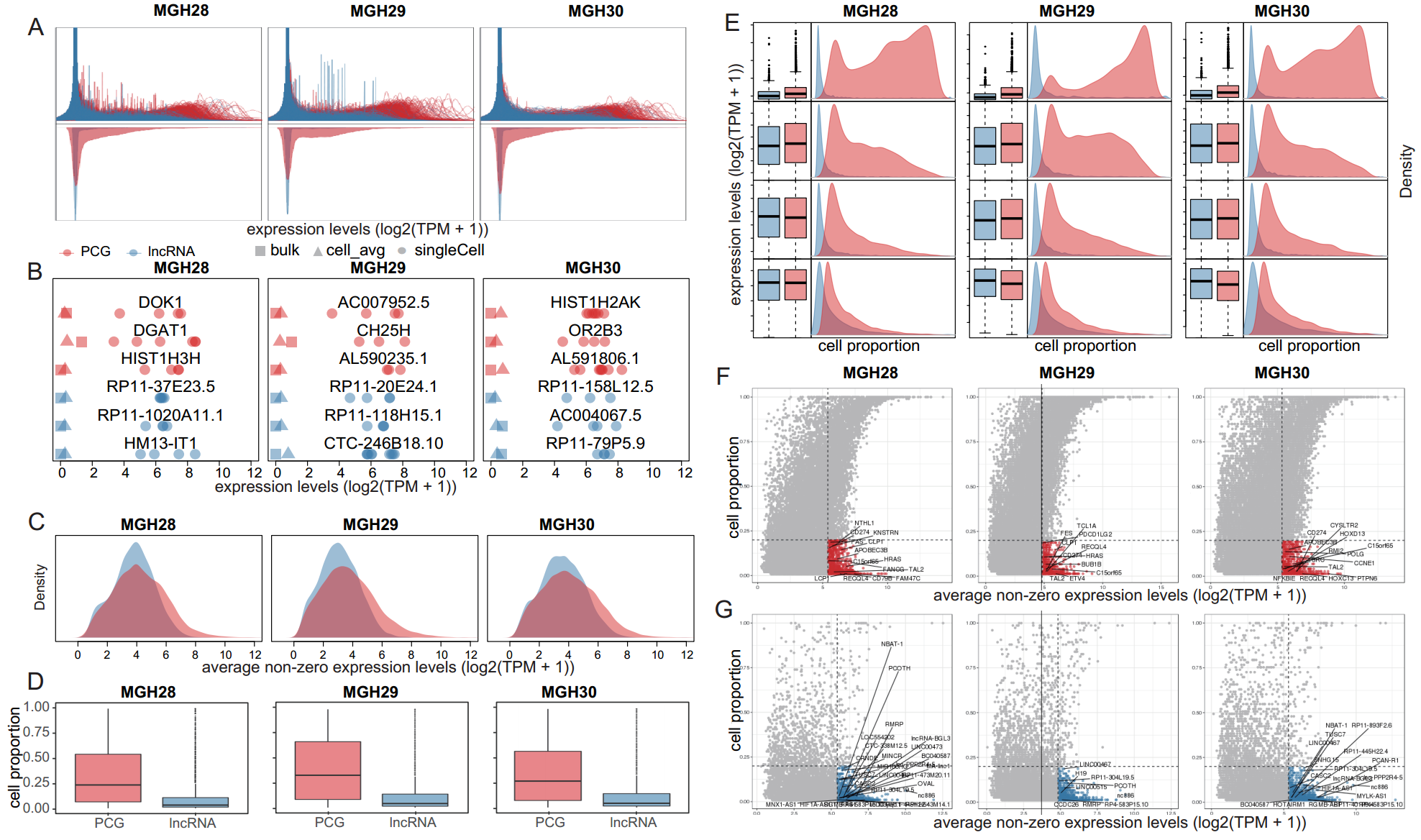


**Figure S2.** The characterization of transcriptional patterns of PCGs and lncRNAs in MGH28, MGH29 and MGH30. (A) Distribution of expression levels of PCGs (lightred lines) and lncRNAs (lightblue lines) in single cells (upper panel) and in bulk samples (lower panel). (B) Examples of PCGs and lncRNAs which were abundant in single cells while rarely detected in bulk sample. (C) Distribution of average non-zero expression levels of PCGs and lncRNAs. (D) Overall distribution of cell proportions of PCGs and lncRNAs among cells from MGH28, MGH29 and MGH30. (E) Distribution of cell proportion for PCGs and lncRNAs grouped by average non-zero expression quartiles of PCGs among cells from MGH28, MGH29 and MGH30; Expression levels of the four groups increased from bottom to top. (F-G) A number of cancer-related PCGs (F) and lncRNAs (G) showed abundant expression in few cells.


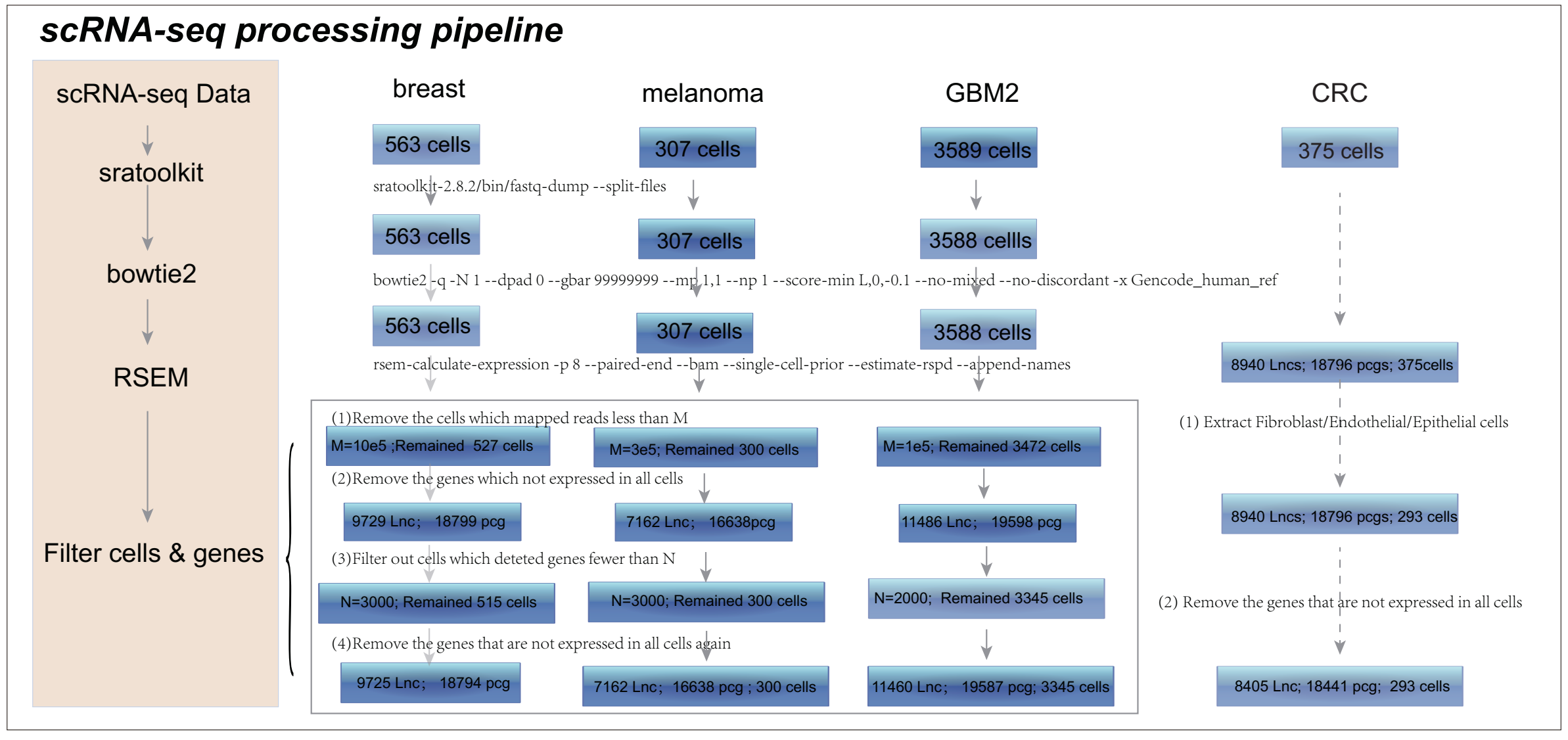


**Figure S3.** The single cell processing pipeline.


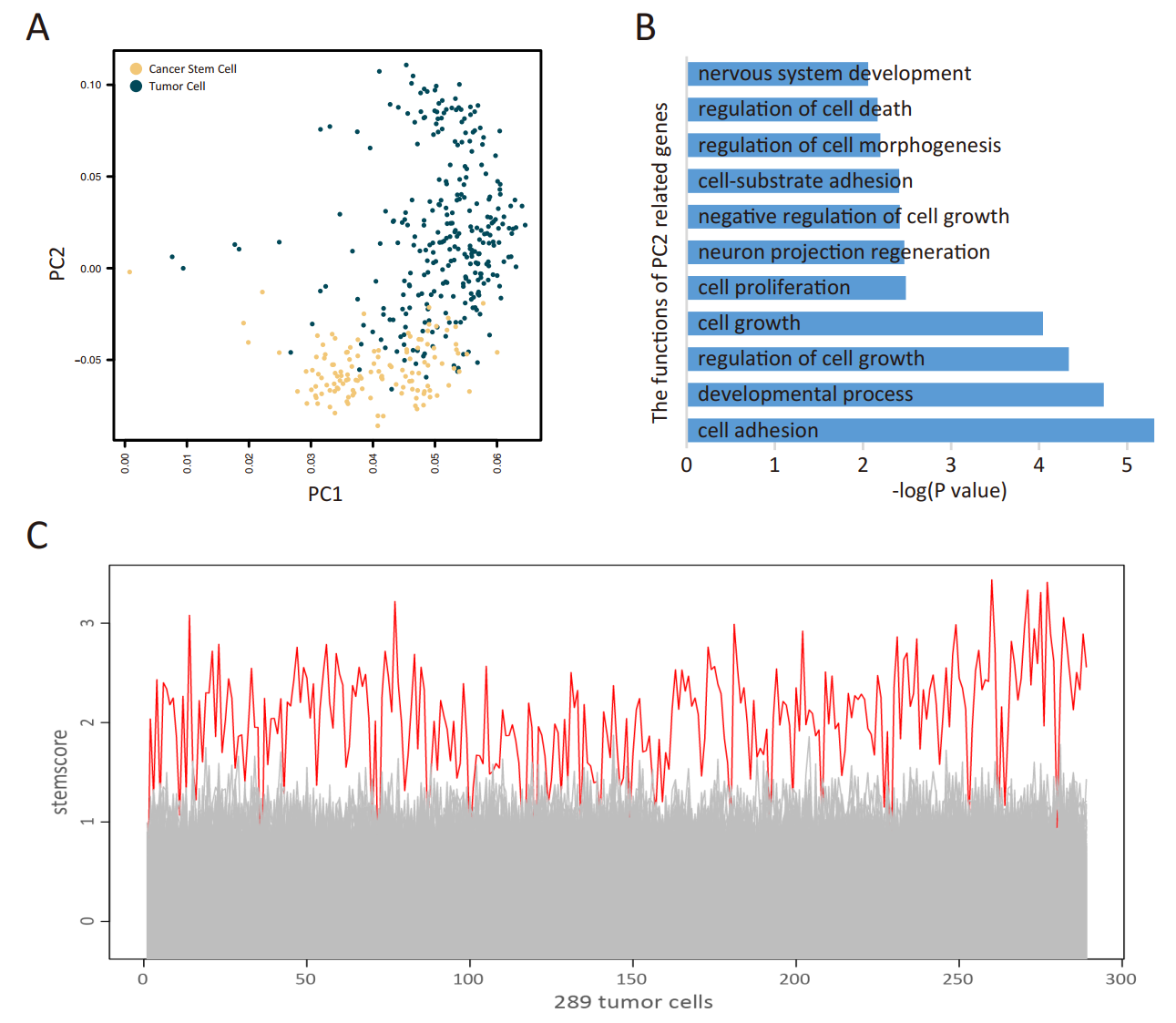


**Figure S4.** The reliability of the 51-gene signature and specific expression patterns of rare genes in GBM stem cells. (A) PCA plot of the 51-gene signature for CSCs and 289 tumor cells. (B) The enriched functions of PC2-related genes. (C) The stemness scores for 289 tumor cells based on 51-gene signature (red line) and 1000 random marker sets (grey lines).


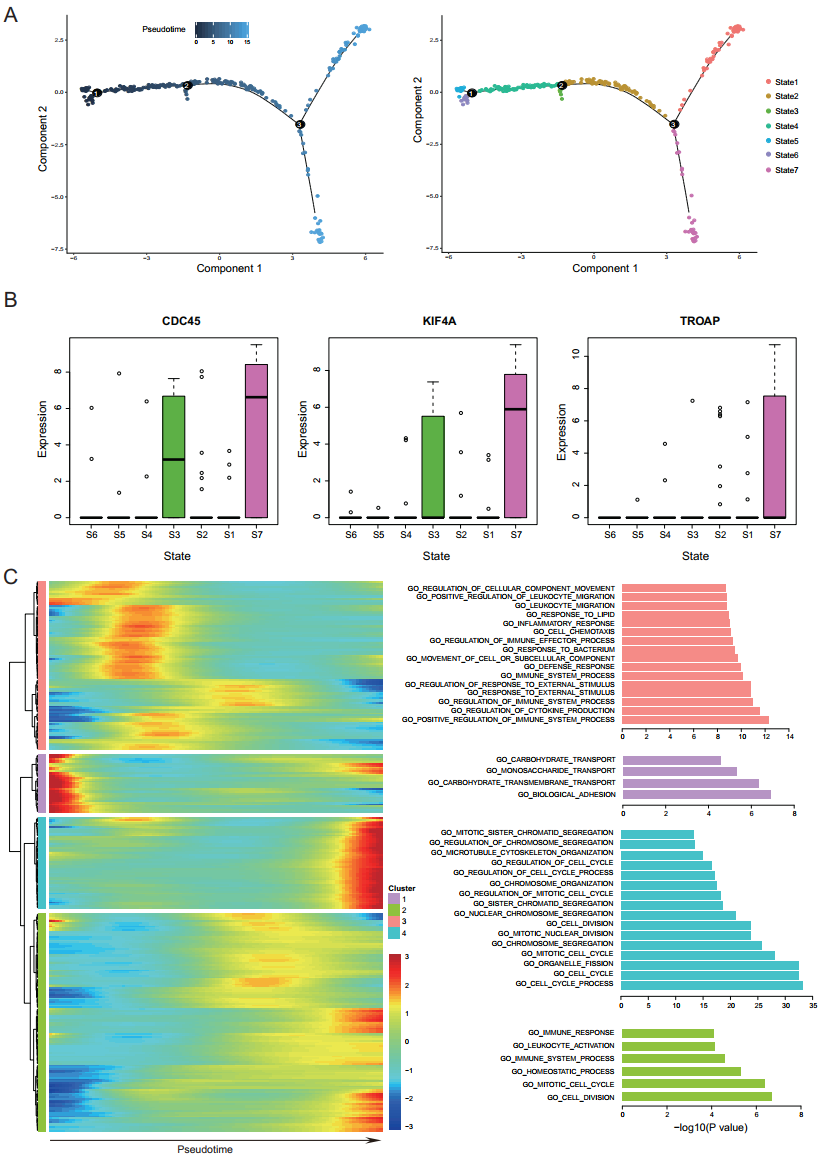


**Figure S5.** The expression levels and functions of rare genes. (A) GBM progression trajectory constructed by Monocle2. (B) The expression levels of 17 cell subset-enriched rare genes in cells with seven states. (C) Heatmap (left) shows the genes expression dynamics during GBM progress. State-specific genes (row) are clustered and cells (column) are ordered according to the pseudotime. Functional gene sets enriched by genes in each cluster (right). The x axis represent the –log10-transformed P values.


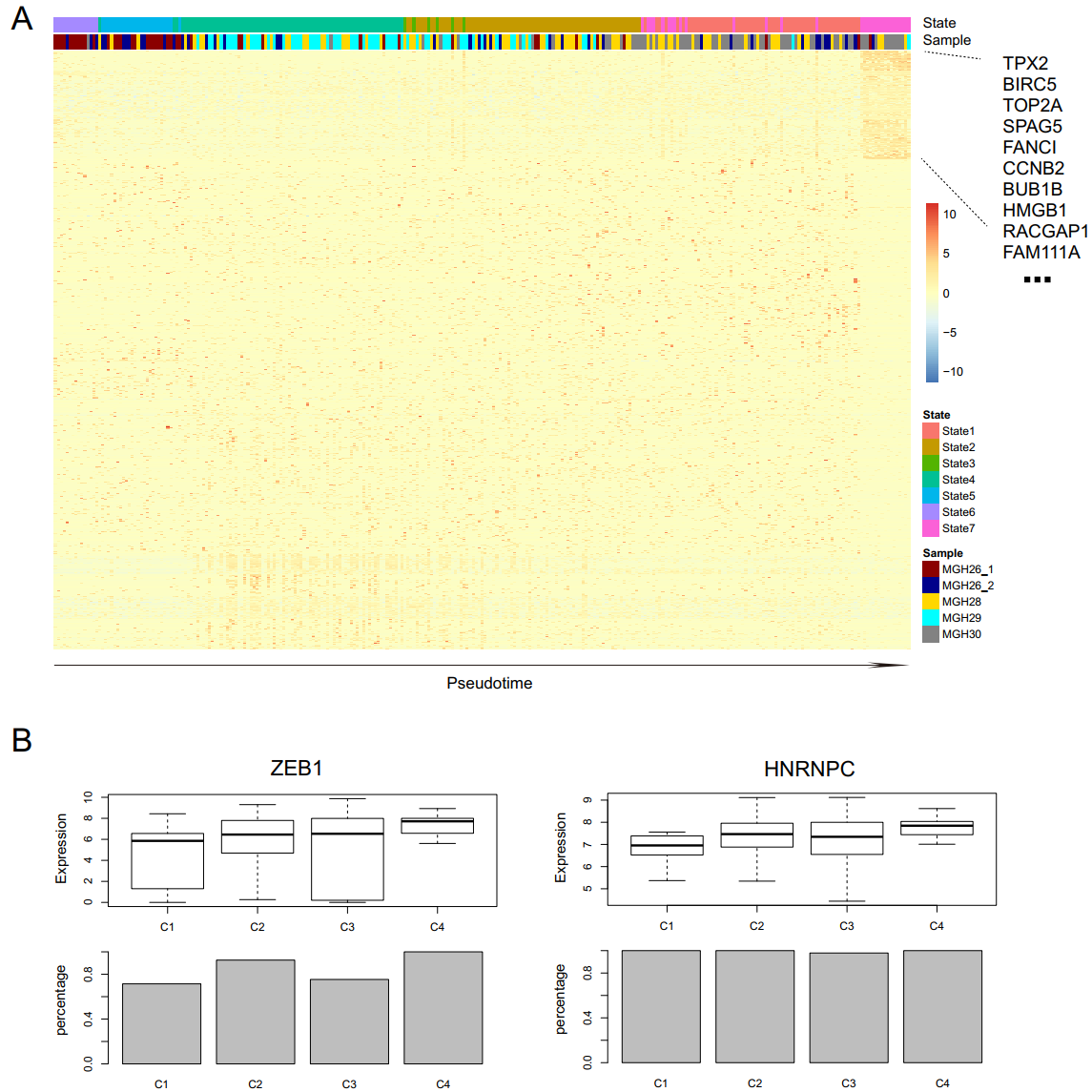


**Figure S6.** Differentially expressed genes and GBM invasion-associated genes. (A) Heatmap shows the differentially expressed genes between a subset of cells and others. Cells are ordered according to the pseudotime. Some highly expressed genes in the subset of cells are shown on the right side. (B) Boxplots (up) showing the expression levels of two GBM invasion-associated genes (*ZEB1* and *HNRNPC*) in four clusters. Barplots (down) showing the percentages of cells that express the two genes in four clusters.
